# Exploiting Magnetic Resonance Angiography Imaging Improves Model Estimation of BOLD Signal

**DOI:** 10.1371/journal.pone.0031612

**Published:** 2012-02-22

**Authors:** Zhenghui Hu, Cong Liu, Pengcheng Shi, Huafeng Liu

**Affiliations:** 1 State Key Laboratory of Modern Optical Instrumentation, Zhejiang University, Hangzhou, China; 2 B. Thomas Golisano College of Computing and Information Sciences, Rochester Institute of Technology, Rochester, New York, United States of America; 3 University of Rochester Medical Center, Rochester, New York, United States of America; Institute of Psychology, Chinese Academy of Sciences, China

## Abstract

The change of BOLD signal relies heavily upon the resting blood volume fraction (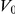) associated with regional vasculature. However, existing hemodynamic data assimilation studies pretermit such concern. They simply assign the value in a physiologically plausible range to get over ill-conditioning of the assimilation problem and fail to explore actual 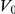. Such performance might lead to unreliable model estimation. In this work, we present the first exploration of the influence of 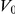 on fMRI data assimilation, where actual 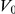 within a given cortical area was calibrated by an MR angiography experiment and then was augmented into the assimilation scheme. We have investigated the impact of 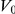 on single-region data assimilation and multi-region data assimilation (dynamic cause modeling, DCM) in a classical flashing checkerboard experiment. Results show that the employment of an assumed 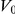 in fMRI data assimilation is only suitable for fMRI signal reconstruction and activation detection grounded on this signal, and not suitable for estimation of unobserved states and effective connectivity study. We thereby argue that introducing physically realistic 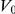 in the assimilation process may provide more reliable estimation of physiological information, which contributes to a better understanding of the underlying hemodynamic processes. Such an effort is valuable and should be well appreciated.

## Introduction

In 1998, Buxton and his colleagues introduced their celebrated hemodynamic model, Balloon model [1]. The comprehensive biophysical model of hemodynamic modulation describes the coupling dynamics from neural activity to observed blood oxygen level dependent (BOLD) signal [1], [2]. It comprises the coupling mechanism of manifold physiological variables, blood flow (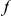), blood volume (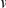), and deoxyhemoglobin content (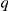), during brain activation. This model then has been extended to include the effects of external inputs on blood flow inducing signal by Friston *et al* [3]. Since its inception, there is a growing interest in assimilating such a model with given sets of fMRI measurements in order to infer physiological parameters and associated states [4]–[9], constrain the activation detection process with classic statistics techniques [10], [11], and extrapolate to similar systems and/or different driving conditions [12]–[16]. Although these works greatly enhance our understanding of the neural systems that mediate specific cognitive processes, they are still kind of problematic in offering reliable inference on the hemodynamic system behaviors.

The query on reliability of estimation primarily comes from the assumption about resting blood volume fraction (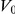) in the assimilation procedure. It has long been noted that BOLD contrast is highly weighted by venous blood content. The change of signal intensity in given region thereby depends heavily on local vessel geometry including capillaries and large veins. The evaluation of model structure also indicates that 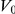 is a leading influence mechanism in driving the model output uncertainty [17]. However, This parameter can not be identified along with other model parameters simultaneously due to the ill-conditioning of the inverse problem. All studies so far have engaged a physiological plausible value 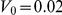 in region of interest (ROI) [3]–[9], [18] or throughout the whole brain [10], [11] to dispel the ill-conditioning problem, instead of investigating actual 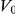. When a voxel includes only brain tissue, the assumption 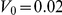 is reasonable [3], [19]. When a voxel is mostly or totally occupied by a vessel or vessels, however, the value might typically be above 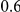 [20]. On the other hand, these voxels that contain large blood content are always more likely to show significant BOLD activation due to the nature of fMRI technique. In this situation, the employment of unrealistic 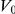 value in data assimilation might produce unreliable model estimation, far straying from physiological reality. The effort of incorporating actual vascular information of voxels into the fMRI data assimilation therefore should be well appreciated.

In this study, we presented the first attempt to exploit actual resting blood volume fraction in assimilation procedure. The actual 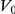 is derived from the segmentation of the vein in the MR angiography (MRA), then augmented into the existing assimilation schemes. As physical realistic 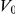 is adopted in assimilation process, more reasonable inference about hemodynamic behavior can thus be expected. We will illustrate the efficacy of the combinative approach on single-region data assimilation and multi-region data assimilation.

This paper is organized as follows. We first simply review the hemodynamic Balloon model and its formulation that forms the basis of data assimilation, then describe the derivation of 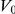 from MRA images. The impacts of actual 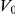 on states forecast and parameter estimation are presented in terms of data assimilation and dynamic causal models subsequently.

## Methods

### Hemodynamic Balloon Model

The original hemodynamic Balloon model consists of three subsystem linkings: (1) neural activity to changes in flow; (2) changes in flow to changes in blood volume and venous outflow; (3) changes in flow, volume and oxygen extraction fraction to changes in deoxyhemoglobin (dHb). It describes the dynamic intertwinement between the blood flow 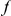, the blood venous volume 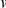 and the veins dHb content 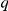, which can be given as the following [1], [3]:
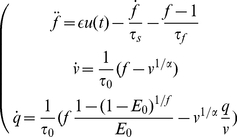
(1)where 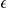 is neuronal efficacy; 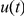 is the neuronal input; 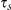 reflects signal decay; 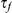 is the feedback autoregulation time constant; 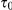 is the transit time; 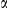 is the stiffness parameter; and 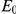 denotes the resting oxygen extraction fraction. All variables are expressed in normalized form, relative to resting values. The input-state-output system is represented by nonlinear equations of a series of physiological states. Equation (1) has a second-order time derivative, and we can write this system as a set of four first-order ordinary differential equations (ODEs) by introducing a new variable 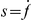. Although the Balloon model is enhanced somehow afterwards [21]–[23], the model structure analysis shows that the original model is sufficient to account for the hemodynamic response in sparse, noisy fMRI measurement [11], [17].

**Figure 1:**
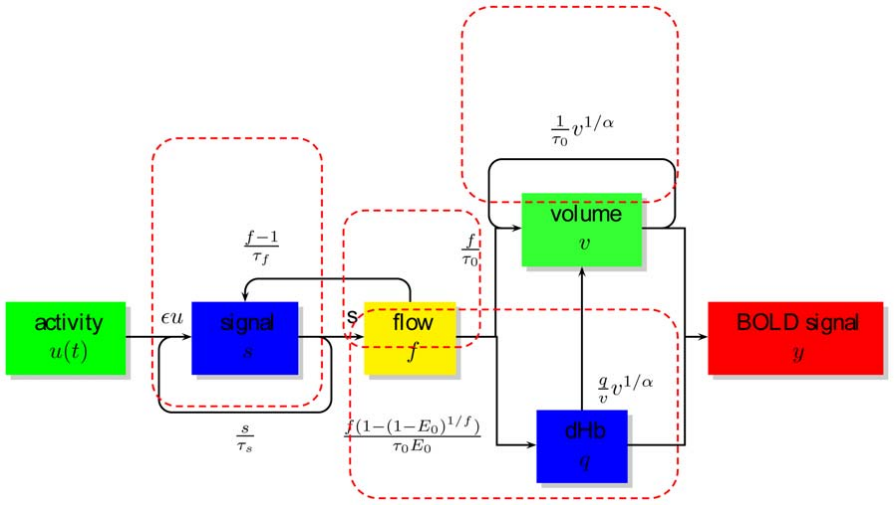
Schematic illustration of the hemodynamic Balloon model.

Furthermore, the BOLD observation can be expressed as:
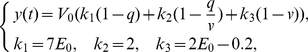
(2)appropriate for a 1.5 Tesla magnet [1]. 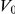 is the resting blood volume fraction, which may vary across brain regions and across subjects. The model architecture is summarised in Figure 1.

For any given combination of parameters 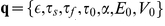 and neuronal inputs 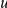, equations (1) and (2) produce a predicted BOLD response. They form the basis for fMRI data assimilation from the measured dataset. Note that parameter 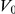 can not be identified along with other parameters simultaneously, but only their product is admitted. Up to now, all existing efforts have circumvented the ill-conditioning nature by imposing a physiological plausible value 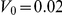 [4]–[8], [10]–[16]. However, the usage of this parameter value is expedient so that the assimilation problem can be solved. Since the change in the BOLD signal depends heavily on 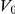, unrealistic 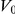 may lead to unreliable model parameter estimation. In addition, the stiffness parameter 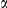 shows a marginal influence to the system output variance, and it can be fixed within its physiological reasonable range (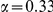 here) without significant loss of information in data assimilation processing [8], [10], [11], [17].

### Derivation of 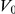 from MR Angiography Image

In hemodynamic model, 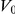 is defined as the venous volume of blood present in a voxel. It represents the ratio occupied vessels with sizes ranging from capillaries to large veins that all contribute to fMRI measurements in the area [1], [2]. Typical resting value in brain tissue which only contains capillaries is around 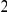 per cent. When a vessel or vessels are present in a voxel, local blood volume will dramatically rise. The value in large vessel region is typically above 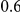. The presence of large vessel is expected to make 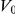 inhomogeneous. Fortunately, large blood vessel are accessible by MR angiography (MRA) imaging.

Consider that 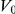 in a voxel consists of two different derivative components, constant tissue blood volume component 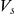 and varied large blood vessels component 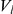:
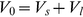
(3)

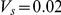
 is small-vessel blood volume (including capillaries and small postcapillaries). 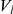 is blood volume of large blood vessels (veins and venules). It is associated with draining veins, and spatially varies across different brain areas in general. In this study we made use of high-resolution time-of-flight magnetic resonance angiography (TOF-MRA) scanning to accurately locate the blood vessels in the brain. The principle of TOF-MRA imaging is based on the enhancement of the signal of dynamic blood flow and the suppression of the signal of static tissues. The resolution of the TOF-MRA image was 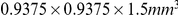 (intensity range [0,1425]), which was much better than that of the fMRI image (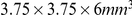, in this study). All images were collected in the same field of view (FOV). In TOF, veins usually bear higher signal intensities than the surrounding tissues, thus making the segmentation of major veins feasible and reliable. It is practicable to downsample the fine vasculature information to coarse fMRI scale in order to obtain the estimation of regional 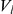 at given fMRI voxels. We therefore attempted to combine the MR angiography image and the fMRI image for uncertainty reduction in data assimilation.

SPM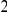 program (Wellcome Department of Cognitive Neurology, http://www.fil.ion.ucl.ac.uk/spm) was used for our data pre-processing, voxel by voxel. Each fMRI volume was realigned to the first volume, and created a mean of the realigned data. The mean functional image then was upsampled to the resolution of the MRA. The MRA image was coregistered (Estimation and Reslice in SPM software) to the resultant upsampled mean image with linear interpolation. For high SNR TOF images, we performed the segmentation by simple thresholding. In the experiment, the segmentation threshold was set to 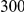 by simple visual guidance. After the vascular segmentation, we can obtain the large blood vessel composition (i.e. 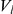) of each voxel in EPI images. Moreover, an isolated voxel with intensity higher than 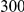 was considered as noise and was therefore excluded from the calculation of 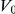. Since the MRA image has a much higher spatial resolution than fMRI image (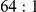 in this study), the large vessel fraction 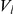 of each voxel was expressed as:
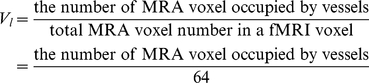
(4)


Combined with the small-vessel fraction 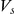, the total blood volume fraction of each voxel in fMRI image was expressed:
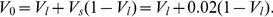
(5)


The first term represents the volume of blood from large vessels in a voxel, the second term is the blood volume fraction in the remaining brain tissue. In this sense, MRA can be thought of as an indirect, physical measurement of 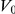, and can be treated as ‘true’ 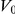 value. 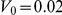 is a special case of the formulation when large vessel does not exist in this voxel.

### Experiment and Data Preprocessing

The participant provided written informed consent before beginning the experiment which was approved by the Health Sciences Research Ethics Committee of Zhejiang University. Functional images were acquired on a 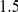-Tesla scanner (Marconi EDGE ECLIPSE) using a standard fMRI gradient echo echo-planar imaging (EPI) protocol (TE, 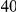 ms; TR, 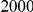 ms; flip angle, 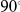; NEX, 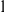; FOV, 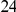 cm; resolution, 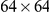 matrix). Sixteen contiguous 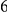-mm-thick slices, 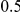-mm-intervals were acquired to provide a coverage of the entire brain. Foam padding was used to limit head motion within the coil.

Before functional imaging, a high-resolution, three dimensional, spoiled gradient recalled at steady state anatomic image was collected (TE, 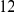 ms; TR, 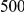 ms; flip angle, 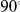; NEX, 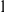; slice thickness, 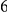 mm; gap, 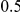 mm; FOV, 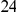 cm; resolution, 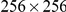 matrix) for anatomic localization and co-registration. Furthermore, a high-resolution angiography image was also collected for segmentation (TE, 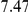 ms; TR, 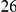 ms; flip angle, 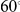; NEX, 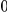; slice thickness, 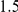 mm; FOV, 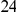 cm; resolution, 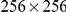 matrix).

Block design experiment was performed in this study. The subject was presented with classical flashing checkerboard pattern when scans were acquired. Activation maps (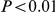) were generated with the SPM software package (Wellcome Department of Cognitive Neurology, http://www.fil.ion.ucl.ac.uk/spm), which used a General Linear Model approach to detect regions with significant response during the task.

**Figure 2:**
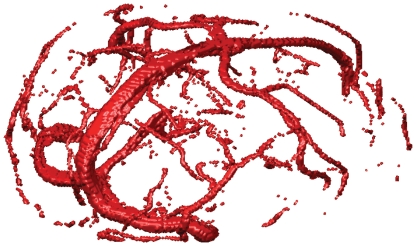
The vasculature of one subject.

The Time-Of-Flight MR Angiography (TOF) image was segmented to extract the major veins in the brain. In TOF, veins usually bear higher signal intensities than the surrounding tissues, which makes the segmentation of major veins easy. For the high signal-noise-ratio (SNR) TOF images, we performed the segmentation by simple thresholding. The selection of threshold could be accomplished manually. In our work, we segmented veins by thresholding because of the high quality of the TOF image. Figure 2 presents an example of CBV imaging segmented from one subject. After thresholding, a de-noise step such as an opening operation, could be executed in order to eliminate some isolated noises. Once the segmented vasculature was obtained, we need to further transfer the information of vein position to the fMRI data. This requires that we registered/aligned the TOF to the fMRI image. Since the subject of the two images was the same patient in a short period, multi-modal rigid registration was enough to perform the task. We chose the classic mutual information as the similarity metric to do the 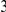
D registration in the physical domain.

**Figure 3:**
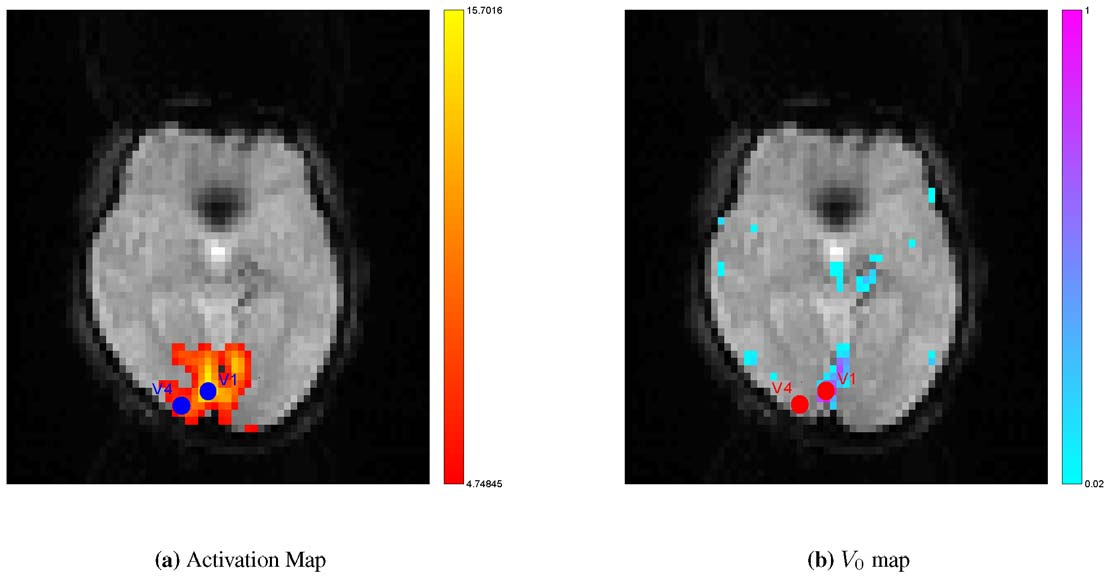
Regions of interest. Because BOLD contrast is highly weighted by venous blood content, activation areas often overlap with large vein regions. Two regions of interest (ROIs) were selected from visual cortex according to activation detection (warm color) and vascular information (cool color). The spatial resolution of venography map was downsample to identical with that of fMRI image. (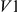: Primary visual cortex; 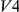: Visual area 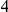).

After finishing the segmentation and registration, we obtained corresponding brain blood volume in the voxel. The actual 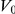 was then augmented into existing assimilation schemes [8], [11], [24]. In this study, two ROIs were selected from the greatest activation locus of primary visual cortex (V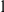) and V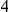 (Figure 3). We defined the clusters based on edges, but not included corners, so that the voxel had 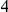 neighbors (in the same slice). Figure 3 clearly shows that the activation area always overlap with the regions of large blood content. The spread activation along large veins in response to experimental stimulus could be observed. Actual 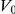 was equal to 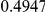 in V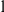 locus due to the presence of large veins (Figure 3, right), and 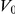 was still 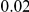 in V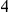 locus because of the absence of large veins. The final time series were extracted by averaging the time series of 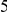 voxels.

## Results

### The Impact of 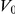 on Single-region Data Assimilation

In this study, as a demonstration, we chose V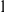 as region of interest. We estimated the state functions and model parameters (Figure 3). Since there are not large veins in V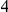 area, this approach makes no difference in this area. For the sake of simplicity, we assumed a constant neural parameter 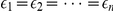 throughout all trials, where 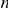 denotes trial number. The estimation scheme is formally identical to that in [24]–[26].

**Figure 4:**
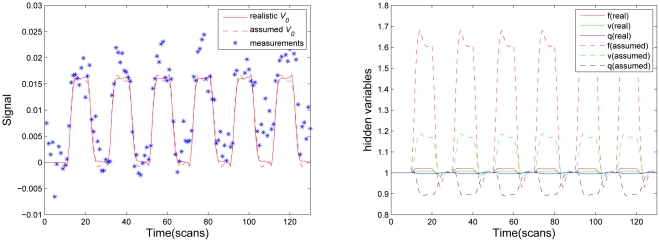
Estimated BOLD signal (Left), and reconstructed physiological states (Right) from the greatest activated locus in primary visual cortex (V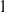). For comparative purpose, model estimation was also performed with a typical assumed 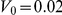. Real 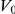 value is 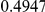.

Figure 4 shows reconstructed BOLD response (left) and underlying physiological states (right) in the greatest activation locus of primary visual cortex (Figure 3) with actual 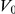, given as solid line. As a comparison, we also evaluated the estimated BOLD response and physiological states with assumed 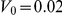 value, which was widely employed in previous studies [3]–[8], [10]–[16], [18], given as dash line in Figure 4. We found that two different 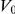 values produced very similar BOLD estimates (Figure 4, left), only tiny discrepancy in post stimulus undershoot stage could be found. Nevertheless, a significant distinction was observed in reconstructed physiological states between two values (Figure 4, right). Though the experimental stimulus induced a puny change in the blood flow 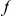, the blood venous volume 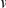 and the veins dHb content 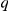, the approach used assumed 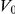 deduced a substantial change during task due to magnifying effect of large blood content. This implied that the presence of large veins in an activated area contributed excess signal in this area. The change of BOLD signal in this area mainly derived from the large-vessel signal, not from the multiple physiological states, namely, not from the experimental related neuronal activity. Since statistical inference essentially is grounded on the amplitude of BOLD response, this area may surely be considered active in statistic analysis of the BOLD signal change, though it is absent at the response efficacy elicited by neuronal activity. This explains why the employment of assumed 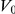 in detection process still could generate very similar activation map with those obtained from classic linear model [10], [11]. The same difference also can be found in estimated model parameters (Table 1). Specially, neuronal efficacy 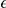 is 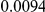 with actual 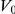 derived from MRA image, while 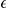 is 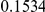 with assumed 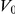 value. Assumed, underestimated 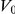 substantially overestimates the neuronal efficacy parameter, 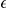. Since parameter 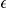 reflects the efficacy with which neuronal activity causes an increase in signal, we argue that the estimated efficiency parameter 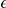 in each voxel might be a good index to sign actual activation level.

**Table 1:** Estimated model parameters with true value (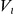) and typical assumed value (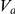) in the greatest activated locus of primary visual cortex (V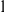).

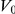	Model Parameters				
	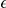	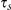	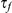	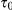	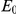
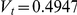	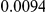	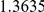	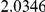	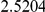	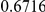
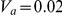	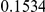	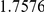	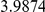	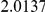	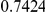

### The Impact of 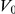 on Dynamic Causal Models (DCM)

Dynamic causal modeling (DCM) has been introduced as a generic method to explore effective connectivity from the hemodynamic observations [12], [27]. Apart from Balloon model, this model additionally embeds a neurobiological modelization of the dynamic interactions among brain areas into the hemodynamic models in these areas, and it can be regarded as an extension of hemodynamic model from single region to covering multiple regions. Single-region data assimilation supposes that extrinsic experimental input consistently accesses all brain regions, whereas DCM designs that inputs produce responses in two different ways: extrinsic influence from sensory input and intrinsic influence from interaction regions. As uncertain 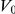 makes the greatest impact on estimates of neuronal efficacy parameter 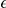 in hemodynamic model, it is interesting to investigate the effect of 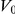 on DCM.

**Figure 5:**
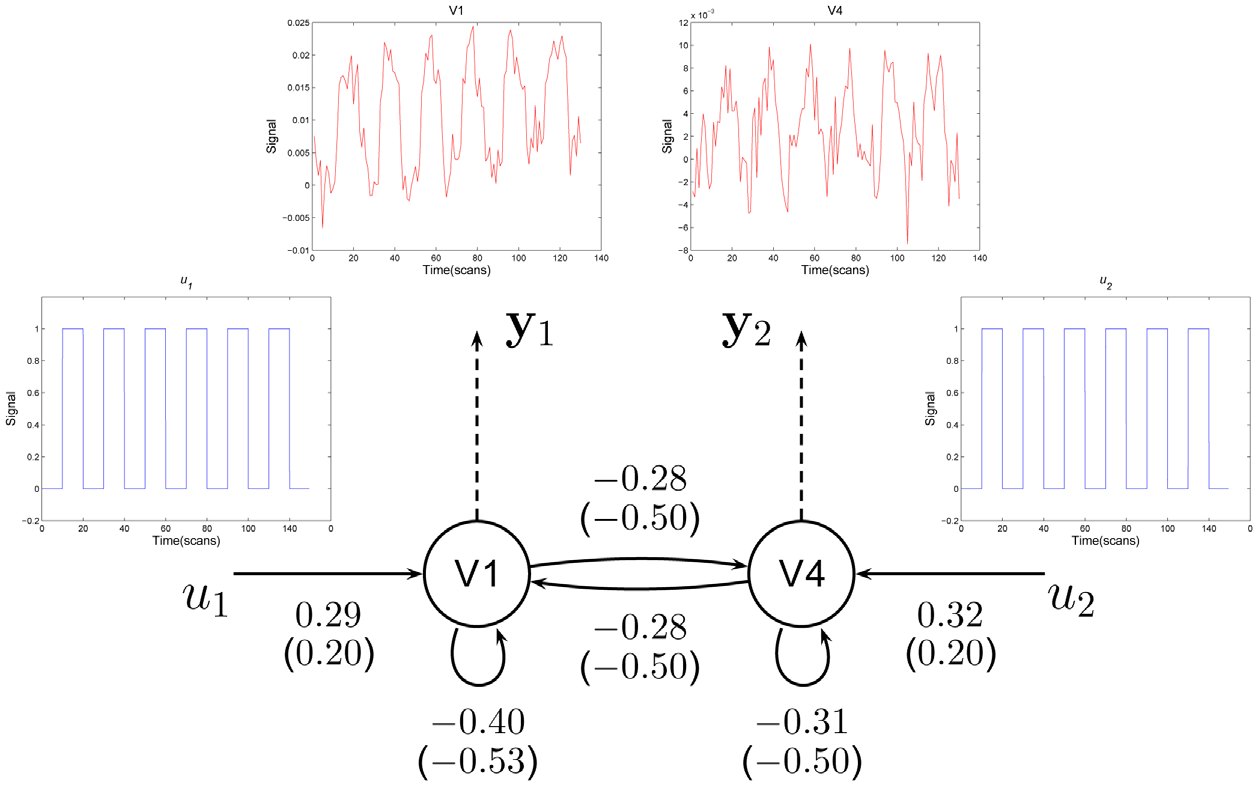
Results of a two-node DCM analysis applied to the flashing checkerboard experiment. The coupling parameters calculated with actual 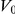 are shown alongside the corresponding connections. The values in brackets are parameters estimated with assumed 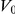. 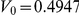 in visual area V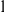, 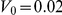 in V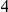 and assumed 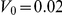 in two areas. 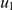 and 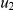 represent external inputs into the system; 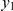 and 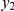 are the hemodynamic observations and arrows indicate connections.

In this study, as an example, two regions were selected using maxima of activation map to construct the hierarchical system. The system architecture was shown in Figure 5. The two maxima were located in visual area V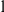 and V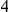. Region-specific time series comprised all neighbor voxels of each maxima location (a total of 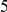 voxels). The location is shown in Figure 3. The system describes a simple hierarchy of forward connections where two primary motor regions influence each other, and can be expressed as the following
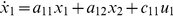
(6)

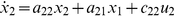
(7)where 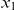
(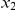) is neuronal dynamics in V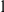 (V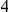); 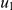 and 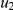 represent external inputs into the system; 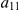
(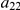) represents the inner connectivity within the region in the absence of input; 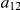
(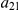) encodes the fixed inter-regions connectivity in the absence of input; 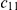
(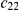) embodies the extrinsic influences of input on neuronal activity.

Equations (6) and (7) then were appended into the states vector [8], [11]. The measurement vector was expanded to include two observations in the two areas as well. Two inputs corresponded to a 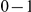 quarewave function for the occurrence of experimental stimulus (Figure 5). The outputs of the system are two time series from two regions. The estimation scheme employed for DCM is formally identical to that in previous studies [8], [11].

The results of this analysis are presented in Figure 5. The connections are shown as directed black arrows with the coupling parameters calculated with actual 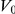 alongside. The values in brackets are parameters estimated with assumed 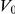. 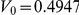 in visual area V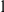, 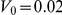 in V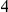 and assumed 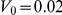 in two areas. As expected, the significant difference in connectivity parameters with actual 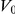 and assumed 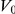 can be found (Figure 5). The fixed connectivity from V
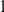
(V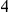) to V
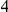
(V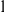) is 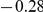 while considering the contribution of vessels, whereas the value is 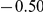 while the effect was discounted.

From the above analysis, the employment of an assumed 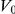 in the hemodynamic data assimilation seems to be only suitable for fMRI signal reconstruction and activation detection grounded on this estimated signal, not for effective connectivity study that by means of estimated neuronal activity (e.g. 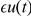) makes inference about the coupling among brain areas and how that coupling is influenced by changes in experimental context. Due to the regulation of resting blood volume fraction (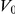), in fMRI imaging, large BOLD signal changes are often associated with large draining veins, while tissue areas have low BOLD signal changes. These results suggest that the impact of 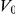 on fMRI data assimilation should be considered. Actual 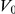 should be investigated or these areas that are dominated by large veins should be excluded in the region-specific analysis.

## Discussion

This work is principally concerned with an important but long ignored issue in previous efforts on the hemodynamic model – the influence of resting cerebral blood volume fraction 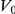. Previous studies postulated a physiologically plausible value 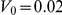 in assimilation procedure to handle ill-posedness of the problem, as opposed to exploring true BVF. This practice may lead to inaccurate results. In this study, instead of arbitrary assignment, we propose a combinative approach that supplements realistic 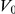 derived from MR angiography (MRA) image into an existing hemodynamic assimilation scheme to achieve more reliable model estimation. We find that 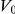 has a complicated influence on assimilation results. Though arbitrarily assigned 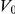 can produce similar BOLD response with realistic 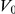, there is significant difference in reconstructed physiological states and estimated model parameters, indicating that the application of these parameter estimated by assumed 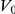 should be justified and interpreted with caution [7], [28]. Moreover, as uncertain 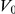 value leads to larger deviation in estimated efficacy parameters 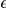 than that in other parameters, we also have investigated the influence of 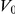 on dynamic causality modeling which estimates connectivity in different brain regions by means of 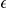 estimates. Not surprisingly, 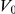 has also considerable impact on the evaluation of brain connectivity. We thereby argue that introducing more realistic 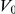 in DCM can provide more reliable estimation of interregional coupling, and assist to acquire a better understanding of brain connectivity that is of considerable interest in neuroimage community recently, such as Human Connectome Project (HCP) in NIH, Brain CONNECT Project in Europe, and National Basic Research Program of China (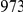) under Grant 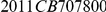.

A possible criticism on this work is to what extent MRA image is able to provide accurate actual cerebral blood volume fraction reflecting BOLD response. Indeed MRA imaging is not a direct, physical measurement of 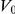. However, as noted, the MRA image has a much higher spatial resolution than a fMRI image (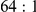 in this study). In this sense, MRA can be thought of as an indirect, physical measurement of large veins component 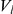 in given areas. Combining with tissue blood volume component 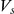, a more realistic 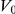 value can be obtained. An imperfect measurement is better than arbitrary assignment without any measurement. As more physical realistic 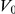 is incorporated into the assimilation procedure, more reliable information of the underlying physiological dynamics is reconstructed, and physiologically more meaningful results may be expected. Another limitation is that few experiments were performed in this study. The main cause is the incorrect but pervasive belief that MRI scans are harmful to health in China. The recruitment of subjects was very difficult. This was even reported recently by Nature [29]. However, our study is mainly concerned with the influence of 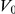 on assimilation, and to discuss the importance of introducing actual 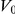 information into the assimilation process. The results clearly illustrate our intention. We therefore believe that more experiments is not necessary. Despite these limitations, we argue that such a effort is valuable and should be well appreciated, in particular, while nearly 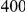 studies on hemodynamic data assimilation have been reported every year [30]. Currently, we are trying to experimentally verify that augmenting more realistic 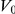 derived from MRA imaging into assimilation process will provide more accurate states forecast and parameter estimation.
